# Warming Reduces Carbon Losses from Grassland Exposed to Elevated Atmospheric Carbon Dioxide

**DOI:** 10.1371/journal.pone.0071921

**Published:** 2013-08-19

**Authors:** Elise Pendall, Jana L. Heisler-White, David G. Williams, Feike A. Dijkstra, Yolima Carrillo, Jack A. Morgan, Daniel R. LeCain

**Affiliations:** 1 Department of Botany and Program in Ecology, University of Wyoming, Laramie, Wyoming, United States of America; 2 United States Department of Agriculture – Agricultural Research Service, Rangeland Resources Research Unit and Northern Plains Area, Fort Collins, Colorado, United States of America; 3 Departments of Botany; Ecosystem Science and Management, and Program in Ecology, University of Wyoming, Laramie, Wyoming, United States of America; 4 Faculty of Agriculture and Environment, University of Sydney, Sydney, New South Wales, Australia; University of Illinois, United States of America

## Abstract

The flux of carbon dioxide (CO_2_) between terrestrial ecosystems and the atmosphere may ameliorate or exacerbate climate change, depending on the relative responses of ecosystem photosynthesis and respiration to warming temperatures, rising atmospheric CO_2_, and altered precipitation. The combined effect of these global change factors is especially uncertain because of their potential for interactions and indirectly mediated conditions such as soil moisture. Here, we present observations of CO_2_ fluxes from a multi-factor experiment in semi-arid grassland that suggests a potentially strong climate – carbon cycle feedback under combined elevated [CO_2_] and warming. Elevated [CO_2_] alone, and in combination with warming, enhanced ecosystem respiration to a greater extent than photosynthesis, resulting in net C loss over four years. The effect of warming was to reduce respiration especially during years of below-average precipitation, by partially offsetting the effect of elevated [CO_2_] on soil moisture and C cycling. Carbon losses were explained partly by stimulated decomposition of soil organic matter with elevated [CO_2_]. The climate – carbon cycle feedback observed in this semiarid grassland was mediated by soil water content, which was reduced by warming and increased by elevated [CO_2_]. Ecosystem models should incorporate direct and indirect effects of climate change on soil water content in order to accurately predict terrestrial feedbacks and long-term storage of C in soil.

## Introduction

Models predict declining C sequestration in the coming century [1], [2], [3] as ecosystem respiration (R_eco_) is preferentially stimulated over ecosystem photosynthesis (P_eco_), but experimental tests for these predictions are lacking [4]. Experimental manipulations of single global change factors have greatly improved our understanding of ecological processes that regulate C exchange [5], [6]. Photosynthesis is stimulated by elevated [CO_2_] due to increased biochemical forcing and improved water use efficiency [7], but the magnitude of these mechanisms varies both within and across ecosystems [8]. Warming has been shown to increase, decrease, and have no effect on C assimilation [6] – responses that are tied to enzymatic reaction rates, plant photosynthetic acclimation, potential changes in growing season length, and resource availability. On a physiological level, R_eco_, composed of both autotrophic (R_a_) and heterotrophic (R_h_) respiration, responds more strongly to temperature than does photosynthesis [9], underpinning model predictions that ecosystem C storage will gradually decrease in a future warmer world [1], [10]. Multifactor climate change experiments are needed to test the predictions of global C cycle models and identify the strength of interactive effects on ecosystem C uptake and loss [4].

Recent modeling and meta-analysis studies suggest that the combination of warming and elevated [CO_2_] will increase biomass and soil respiration in grassland [11], [12], but this does not address what mechanisms underlie the responses. Furthermore, only six experiments in the meta-analysis combined these treatments in natural ecosystems, and of those, just one reported measurements of R_h_, which is a key determinant of long-term climate – C cycle feedbacks. Biomass and respiration responses to elevated [CO_2_] are known to be mediated by indirect effects of soil water enhancement [8], [13], whereas warming-induced drying may counteract the effects of moisture on C cycling. These emergent, ecosystem-level properties are not well represented in meta-analyses or simulation models, because virtually no experimental data exists to validate the models.

Since 2006, we have conducted a global change experiment in a temperate semi-arid native grassland in southeastern Wyoming, USA to study the combined impacts of elevated [CO_2_] and warming on ecosystem C dynamics and C balance. The **P**rairie **H**eating **A**nd **C**O_2_ **E**nrichment (**PHACE**) experiment combines a full-factorial manipulation of these global change conditions and a supplemental irrigation treatment across 25 replicate plots (n = 5 per treatment type). Previous research at PHACE demonstrated that elevated [CO_2_] and warming together enhanced net primary production (NPP), especially in C4 grasses [14], but the gross and net CO_2_ fluxes, and therefore feedbacks to climate change, are still in question.

We hypothesized that soil water availability (SWC) would mediate treatment effects on gross CO_2_ fluxes and R_h_, such that 1) elevated [CO_2_] alone (referred to as the Ct treatment) would stimulate R_eco_ more than P_eco_ due to enhanced R_h_, leading to net C losses, compared to the ambient (ct) treatment [13], [15]; 2) warming alone (cT treatment) would stimulate P_eco_ and suppress R_eco_ relative to ambient conditions, as soil moisture limits R_h_, leading to net C gains relative to ambient conditions [6]; and that 3) elevated [CO_2_] plus warming (CT treatment) would lead to no stimulation or suppression of R_eco_ or P_eco_ relative to ambient conditions, because SWC is the same in this treatment as ambient [14]. We further hypothesized that CO_2_ fluxes in the irrigated (ct-i) treatment would follow a similar pattern to the elevated [CO_2_] treatment because SWC was manipulated to match that of the Ct treatment.

## Materials and Methods

### Experimental Manipulation and Field Site

The Prairie Heating and CO_2_ Enrichment (PHACE) experiment is located in a northern mixed grass prairie (NMP) ecosystem at the United States Department of Agriculture Agricultural Research Service (USDA-ARS) High Plains Grasslands Research Station in Cheyenne, Wyoming, USA, with full approval by HPGRS management. The vegetation is dominated by the C_3_ grass *Pascopyrum smithii* (Rydb.) A Love and the C_4_ grass *Bouteloua gracilis* (H.B.K) Lag, and other abundant species include the C_3_ grass *Hesperostipa comata* Trin and Rupr., the sedge *Carex eleocharis* L. Bailey, and the sub-shrub *Artemisia frigida* Willd. No protected species were sampled during this research. Mean annual precipitation is 384 mm and mean maximum and minimum air temperatures are 17.5°C in July and −2.5°C in January, respectively. The soil is a fine-loamy, mixed, mesic Aridic Argiustoll.

In 2005, 25 circular plots were established ca. 3.4 m in diameter and surrounded by an impermeable barrier that was buried to 60 cm soil depth. Free-Air CO_2_ Enrichment [16] began in 2006 and elevates [CO_2_] to 600 µmol mol^−1^, and warming began in 2007 with infrared heaters that elevate plant canopy temperatures 1.5 and 3.0°C during the day and night, respectively [17], in a full factorial design with 5 replicates for each of the 4 combinations (ct, ambient CO_2_ and ambient temperature; cT, ambient CO_2_ and elevated temperature; Ct, elevated CO_2_ and ambient temperature; and CT, elevated CO_2_ and elevated temperature). Five plots were exposed to ambient CO_2_ and temperature and received periodic irrigations to maintain soil water content (SWC) similar to that in elevated CO_2_ plots (referred to as ct-i in figures). These plots received 20-mm irrigations five times in 2007 (7 June, 20 June, 11 July, 21 September, and 15 November), three times in 2008 (26 June, 18 July, and 19 September), three times in 2009 (17 July, 10 August, and 28 September), and three times in 2010 (1 July, 22 July, and 20 August).

### Continuous Measurement of Soil Moisture

Within each plot, volumetric soil moisture was measured at 10 and 20 cm soil depths (EnviroSMART probe; Sentek Sensor Technologies, Stepney, Australia) and logged (via CR10X data loggers; Campbell Scientific, Logan, Utah, USA) hourly from 2007–2010. We calculated soil water content (SWC; cm H_2_O) for the 0–10 cm and 10–20 cm soil depths and summed the amounts to arrive at SWC for the upper 0–20 cm of the soil profile.

### Ecosystem C Flux Measurements

We used a static chamber method [18] to measure ecosystem C fluxes on ca. 50 days between May 2006 and October 2010 through a combination of mid-day and diurnal sampling campaigns. Flux measurements occurred every 2–4 weeks during the growing season, with diurnal sampling campaigns at approximately 6 week intervals and midday campaigns during the intervening periods. Both net ecosystem exchange (NEE) and ecosystem respiration (R_eco_) were measured, and ecosystem photosynthesis (P_eco_) was calculated according to P_eco_ = NEE − R_eco_. For each diurnal, NEE and R_eco_ were measured 5 times over the course of 24 hours (at ca. 0400, 0900, 1300, 1600, and 2100 hours). We used a Lexan polycarbonate (GE plastics, Pittsfield, MA) chamber fitted with 2 circulating fans, a Q190 photosynthetically active radiation sensor (LI-COR, Lincoln, NE), and an open-path LI-7500 infrared gas analyzer (LI-COR) for measuring [CO_2_]. Two chambers were used in tandem so that the sampling time period was constrained to ≤2 hours. NEE was measured for 2 minutes, and then an opaque cover was placed over the chamber to block light and eliminate photosynthesis to measure R_eco_ for the next 2 minutes.

Ecosystem CO_2_ fluxes were calculated after applying a correction for water vapor dilution [19]. Comparisons of CO_2_ fluxes between the two chambers on a subset of the plots revealed no significant offset from a 1∶1 line, with a correlation coefficient of 0.96, indicating no chamber bias. For each diurnal field campaign, integrations of daily NEE, P_eco_, and R_eco_ were calculated using linear interpolation between measurement points to calculate hourly C balance, summed over 24-hours and presented as g C m^−2^ day^−1^.

We used simple linear regression to establish scaling relationships between mid-day and daily C fluxes [18] (Figures S1, S2). This allowed us to scale frequent mid-day measurements to daily sums of C uptake or loss (g m^−2^ day^−1^). Because daily NEE cannot be directly estimated from mid-day NEE, we used the equation NEE_daily_ = P_ecodaily_+R_ecodaily_ (where P_ecodaily_ and R_ecodaily_ were first estimated by linear regression). We arrived at growing season (May – October) P_eco_, R_eco_ and net ecosystem production (NEP) values by using linear interpolation to estimate daily net C fluxes for all days between measurement dates and then summing all daily data for each season. This simple gap-filling method to estimate seasonal carbon fluxes allowed us to make nearly simultaneous measurements at 25 plots during 50 campaigns. Our measurements were representative of climatic conditions encountered within the field site. This temporal variability did not interact significantly with the climate change treatments [18], so the summation method does not affect the outcome of statistical tests.

### Heterotrophic Respiration and Soil C

In early May of 2008, we established root exclusion plots, a standard method for separating soil respiration into root and microbial components [20]. We installed root barriers to 25 cm depth and applied glyphosate, a broad-spectrum herbicide, to a small area of each plot. PVC rings 25-cm in diameter were inserted 8-cm deep into the soil and a standard static chamber method [21] was used to analyze CO_2_ efflux from headspace samples collected at weekly to biweekly intervals. Syringes were used to collect headspace air three-four times over a 45 minute period, which were analyzed for CO_2_ by gas chromatography (Varian 3800 gas chromatograph equipped with thermal conductivity and flame ionization detectors, Varian Instruments, Sunnyvale, CA, USA). Soil C concentrations were determined on root-free, acidified (1 N H_3_PO_4_) soil samples collected by coring plots in mid-July with a Costech elemental analyzer (Cernusco, Italy).

### Methodological Limitations

Ecosystem-scale flux measurements are required to quantify net C storage on land, and partitioning the net CO_2_ flux into its gross components of ecosystem respiration and photosynthesis demonstrates the physiological control over C storage. We acknowledge the potential for artifacts associated with our chamber techniques, including short-term light-enhanced dark respiration [22] and offsets between light and dark microbial respiration [23]. While increased recognition of these leaf-level phenomena demonstrates a role in ecosystem-scale C cycling, their contribution to fluxes reported here is estimated to be <10%, and within the error of the measurements, because aboveground biomass is only about 20% of total biomass [14], and ecosystem respiration is contributed by approximately equal parts of soil organic matter decomposition and root respiration.

### Statistical Analyses

We used a repeated measures mixed effects model with CO_2_ level, temperature level, and year to test for main and interactive effects of global change treatments on cumulative annual carbon fluxes (P_eco_, R_eco_, and NEP). To determine whether C fluxes in response to elevated [CO_2_] and irrigation were similar, a single factor ANOVA with irrigation as the main effect was used. When necessary, data were log-transformed to meet assumptions of normality and equal variance (soil C). Throughout the text, we characterize significant results according to P≤0.05 and numerical values are presented with standard error of the mean (SEM).

## Results

**Figure 1:**
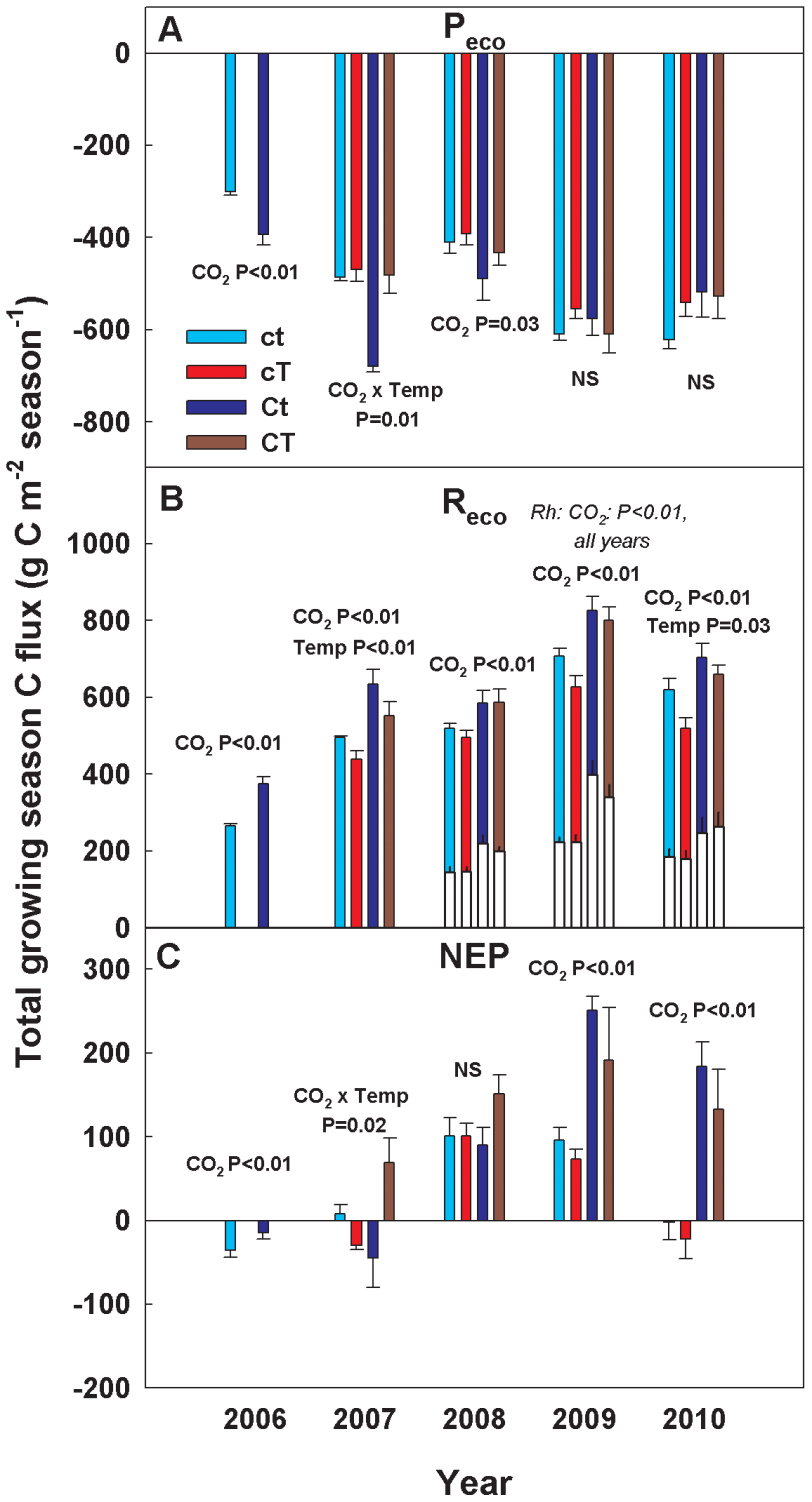
Growing season carbon fluxes in response to global changes. Growing season sums (April–October, 2006–2010) for **A)** gross ecosystem production (P_eco_), **B)** ecosystem respiration (R_eco_) and heterotrophic respiration (Rh) inset white bars, and **C)** net ecosystem production (NEP) for control and global change treatments at the Prairie Heating and CO_2_ Enrichment Experiment in Cheyenne, WY USA. Negative (–) values indicate C uptake and positive (+) values indicate C efflux. Treatment codes are: ct = ambient [CO_2_] and temperature, cT = ambient [CO_2_] and warming, Ct = elevated [CO_2_] and ambient temperature, and CT = elevated [CO_2_] and warming. Statistically significant main and interactive treatment effects (within a given year) along with p-values are indicated (n = 5 for all measurements).

Under present ambient conditions, our data suggest that the semi-arid grassland of the PHACE experiment site ranged from being a slight C sink to a slight source to the atmosphere over 2006–2010; with growing season (April–October) net ecosystem production (NEP, the sum of NEE) losses averaging +33 g m^−2^ over this period (Fig. 1C; positive fluxes indicate mass transfer from the ecosystem to the atmosphere, and negative fluxes indicate mass transfer from the atmosphere to the ecosystem). Elevated [CO_2_] reduced net C uptake in 2006, and caused significantly greater net C losses than ambient in 2009 and 2010 (Fig. 1C; ANOVAR P<0.01 in all 3 years; n = 5). No significant elevated [CO_2_] effects on NEP were observed in 2007–2008. Warming alone never affected NEP, but when combined with elevated [CO_2_], led to significant net C loss in 2007 (Fig. 1C; ANOVAR P = 0.02; n = 5); consistent net C losses were observed from CT treatment from 2007 through 2010.

Elevated [CO_2_] enhanced C cycling by stimulating both gross fluxes, P_eco_ and R_eco_, from 2006–2008 (Fig. 1), years in which both average and below-average annual precipitation were experienced [14]. During these first three years of the experiment, P_eco_ was stimulated by 19–40%, and R_eco_ by 13–42% (Table 1). Beginning in 2009, however, elevated [CO_2_] ceased to stimulate P_eco_ but continued to stimulate R_eco_. This continued stimulation of R_eco_ led to NEP losses that averaged 93 g m^−2^ per growing season (Fig. 1; Table 1). These cumulative CO_2_ losses did not lead to measurable net changes in soil C storage or concentrations during 2006–2010.

**Table 1:** Treatment effects on ecosystem CO_2_ fluxes.

Treatment	Flux	2006	2007	2008	2009	2010
Warming	P_eco_	NA	0.97	0.96	0.91	0.87
Elevated [CO_2_]		1.31*	1.40*	1.19*	0.95	0.84
Elevated [CO_2_]×Warming		NA	0.99	1.05*	1.00	0.85
*Irrigation*		NA	*1.38*	*1.21*	*0.93*	*0.84*
Warming	R_eco_	NA	0.89*	0.95	0.89	0.84*
Elevated [CO_2_]		1.42*	1.28*	1.13*	1.17*	1.14*
Elevated [CO_2_]×Warming		NA	1.12*	1.13*	1.13*	1.07*
*Irrigation*		NA	*1.18*	*1.11*	*0.97^§^*	*0.90^§^*

Ecosystem photosynthesis (P_eco_) and respiration (R_eco_) were measured at the Prairie Heating and CO_2_ Enrichment Experiment during 2006–2010, and are presented as response ratios. Data reflect ratios of a given treatment relative to ambient conditions and are based on cumulative growing season fluxes. Irrigation effects are shown in italics. Statistically significant differences (P<0.05, ANOVA) between core treatments (Warming, Elevated [CO_2_], and Elevated [CO_2_]×Warming) and the ambient treatment within a given year are indicated by an asterisk (*). Significant differences (P<0.05, ANOVA) between Irrigation effects and Elevated [CO_2_] effects are indicated by (*^§^*).

**Figure 2:**
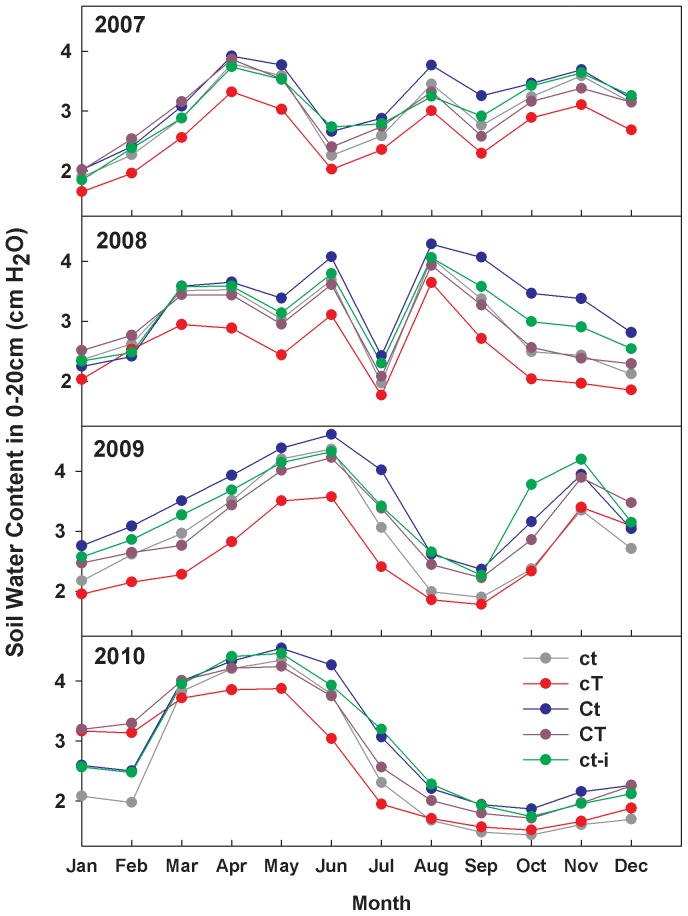
Monthly average soil water content in response to global changes. Mean monthly soil water content (SWC, 0–20 cm) for ambient and global change treatments (2007–2010). Treatment codes are ct = ambient temperature and ambient [CO_2_]; cT = elevated temperature and ambient [CO_2_]; Ct = ambient temperature and elevated [CO_2_]; and CT = elevated temperature and elevated [CO_2_]; ct-i = ambient temperature and ambient [CO_2_]+irrigation.

**Figure 3:**
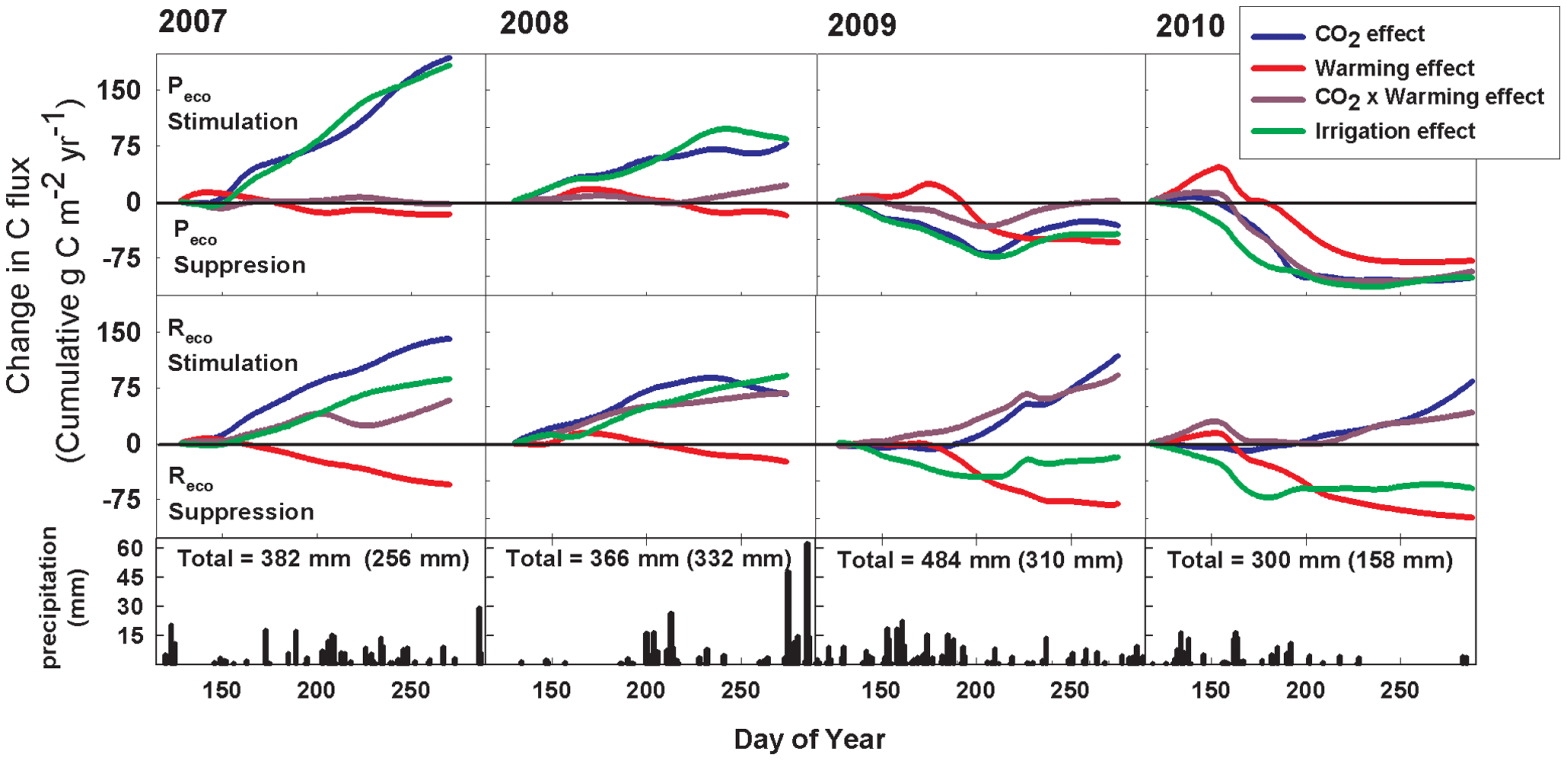
Seasonal patterns of global change effects on C fluxes. Cumulative effects of global changes on gross ecosystem production (P_eco_; g m^−2^ yr^−1^) and ecosystem respiration (R_eco_; g m^−2^ yr^−1^) in 2007–2010 at the Prairie Heating and CO_2_ Enrichment Experiment (PHACE) in Cheyenne, WY USA. Treatment effects are differences from ambient values for each year of the experiment with increases in fluxes expressed as “stimulation” and decreases in fluxes expressed as “suppression.” Data are expressed as cumulative across the growing season with the CO_2_ effect in blue, warming effect in red, CO_2_×warming interactive effect in purple, and irrigation effect in green. Daily precipitation amounts are depicted in the bottom panel. Both annual and growing season precipitation totals (in parentheses) are included for each year.

Throughout the experiment, soil water content (SWC) was increased in elevated [CO_2_] plots on average by 21% during the growing season (Fig. 2), owing to reduced stomatal conductance and transpiration under elevated [CO_2_] [8]. We evaluated the influence of this indirect SWC effect on C fluxes by frequently irrigating an additional set of non-CO_2_ treated plots (n = 5) and maintaining SWC close to that observed under elevated [CO_2_] (Fig. 2). Both the seasonal pattern of daily P_eco_ (Fig. 3) and the annual P_eco_ response were nearly identical in elevated [CO_2_] and irrigated plots in all years of the experiment (Fig. 3; Table 1). Further, the addition of 60 mm of water to irrigated plots did not stimulate P_eco_ in 2009 and 2010 (Table 1, Fig. 3), indicating that water availability under ambient conditions did not limit P_eco_ in these two wetter years. This suggests that stimulation of P_eco_ by elevated [CO_2_] was primarily due to enhanced soil water availability rather than biochemical forcing, and that elevated [CO_2_] alleviates water limitations that might otherwise constrain P_eco_.

Seasonal trends for R_eco_ under elevated [CO_2_] were similar to the shallow irrigation treatment in 2007–2008 (Fig. 3), suggesting that SWC was important in stimulating R_eco_ during those years of average moisture. However, in 2009–2010, the seasonal trends and cumulative fluxes diverged between elevated [CO_2_] and irrigation treatments (Fig. 3; Table 1), suggesting that other mechanisms, in addition to SWC, drove the R_eco_ response to elevated [CO_2_].

Warming suppressed C cycling by reducing R_eco_, especially during dry years of 2007 (P<0.01) and 2010 (P = 0.03) (Fig. 1; Table 1). In 2007, the suppression of R_eco_ by warming alone was sufficient to enhance net C uptake, but when warming was combined with elevated [CO_2_], net C losses were enhanced (Fig. 1; [CO_2_]×temperature interaction, P = 0.02). The warming treatment decreased growing season SWC by 15% on average during 2007–2010 compared to ambient (Fig. 2). Cumulative P_eco_ was not affected by warming (Fig. 1A; Table 1), although it was stimulated early in the wet growing season of 2010 (Fig. 3) when SWC was highest (Fig. 2). This stimulatory effect of warming, however, was quickly reversed as soil water was depleted below levels observed in ambient treatment plots. These opposing responses, within a single growing season, emphasize the role of water availability in mediating C assimilation responses to warming.

Elevated [CO_2_] led to both gains and losses of C (depending on the year), but when elevated [CO_2_] was combined with warming, only net C loss was observed (Fig. 1C). This was driven by consistent enhancement of R_eco_ with elevated [CO_2_] (Fig. 1B) combined with a neutral response of P_eco_ (Fig. 1A). From 2007–2010, R_eco_ was stimulated by an average of 111%, resulting in a net efflux of 259 g C m^−2^ over 4 years under elevated [CO_2_] plus warming, compared to ambient conditions. Compared to NEP under ambient conditions, 545 g m^−2^ of C (237% increase) were lost from the ecosystem with elevated [CO_2_] plus warming during 2007–2010 (Fig. 1C).

We tested for the possibility of enhanced decomposition at PHACE by measuring respiration from root exclusion plots beginning in 2008. Elevated [CO_2_] was observed to stimulate decomposition, or R_h_, by 145% averaged over the last three years of the experiment (Fig. 1B, white bars), but R_a_ (estimated as the difference between R_eco_ and R_h_) was not significantly affected by any treatment. Soil moisture in root exclusion plots was not affected by elevated [CO_2_], and was less affected by warming compared to plots with intact vegetation [24]. Therefore the stimulation of R_h_ by elevated [CO_2_] may have mainly been driven by increased C substrate in the form of dissolved organic C [25] and/or fine root biomass [14].

## Discussion

Our measurements of net CO_2_ exchange (NEE) and its components of ecosystem CO_2_ uptake (P_eco_) and release (R_eco_) in future climate conditions indicated consistent, inter-annual net CO_2_ loss in a semi-arid grassland in response to the combined global changes of elevated [CO_2_] and warming. This result is in contrast to our third hypothesis that the combined treatment would not be different than ambient conditions, and to a recent modeling study indicating net C uptake with elevated [CO_2_] and warming in other grasslands [12]. This positive feedback to climate change arose due to the prolonged stimulation of R_eco_ and the absence of consistent P_eco_ stimulation by elevated [CO_2_], in agreement with our first hypothesis. The main effect of warming was to dampen the CO_2_ loss, particularly in dry years, but P_eco_ was never stimulated by warming, and net C gain never occurred, in opposition to our second hypothesis. While it has been suggested that part of the terrestrial C sink is due to recent warming and lengthening of the growing season [26] our results agree with evidence from climate and remote sensing data [27], suggesting that these changes do not necessarily lead to greater C uptake and assimilation when integrated over the growing season.

Our results indicate that indirect effects of both warming and elevated [CO_2_] on soil moisture strongly affected R_eco_. Soil water availability is an important driver of soil and ecosystem respiration – which are comparable in our ecosystem due to the short vegetation stature – especially in water limited environments [28]. A modeling study suggested that precipitation was the dominant environmental influence over R_s_ in drylands [29], which contrasts somewhat with our findings that added irrigation sometimes stimulated and sometimes suppressed R_eco_ (Table 1; Figure 3). The same modeling study suggests that in simulated future climate, warming will increase the effect of elevated [CO_2_] by additional stimulation of R_s_ [29]. This positive effect of combined warming and elevated [CO_2_] was observed in a mesic herbaceous ecosystem [30]. By contrast, our results suggest warming suppressed the effect of elevated [CO_2_] on R_eco_, probably due to soil drying (Table 1; Figure 2).

The importance of substrate availability in the enhancement of respiration by elevated [CO_2_] was inferred from the larger stimulation of R_eco_ than in the irrigated plots (Figure 3). Stimulation of P_eco_ by elevated [CO_2_] in the initial years of this experiment, and subsequently greater above- and belowground biomass production [14], may have increased substrate availability [25], [31] to stimulate decomposition by an enhanced priming effect [32]. This is consistent with findings from a CO_2_ enrichment experiment in a similar grassland where increased belowground C availability was much more important than soil moisture in stimulating soil respiration [33]. Further, increased labile soil C has often been associated with increased R_s_ in elevated [CO_2_] experiments [13], [34], [35]. If labile C inputs lead to priming of soil organic matter decomposition (R_h_) [13], C losses may be increased with elevated [CO_2_] [15], [36]. In our experiment, increased supply of C substrates belowground from a larger root system also stimulated microbial activity [25], contributing to the enhanced R_h_ associated with elevated [CO_2_]. Our work demonstrates that the considerable stocks of C stored in grassland soils will be vulnerable to future global changes if R_h_ is broadly stimulated by elevated [CO_2_] (Figure 1). However, soil C contents did not change over the 5-year duration of our study.

Warming at PHACE stimulates soil microbial temperature sensitivity at optimal moisture conditions [37], indicating that both elevated [CO_2_] and warming can have the potential to diminish C sequestration in grassland soils. However, drier soils in the field may offset the enhanced temperature sensitivity we observed in the laboratory. The drying effect of warming clearly should be taken into account in modeling studies, some of which still predict enhanced R_h_ with warming, even in dryland ecosystems [29].

We expected to observe more consistent stimulation of C uptake (P_eco_) by elevated [CO_2_], although ample soil moisture availability can suppress the stimulatory effect of elevated [CO_2_] on biomass growth [14]. Variable C uptake is expected in grassland ecosystems, which are characteristically water-limited and experience high interannual variability in precipitation [38]. Warming suppressed the enhancement of P_eco_ by elevated [CO_2_] in 2007 with a significant interaction (Figure 1), probably due to reduction of moisture availability (Figure 3). Our experiment indicated relatively small effects of climate change on gross C uptake in comparison to gross C losses, indicating a continued need to improve understanding of respiratory process responses to climate change [39]. These findings underscore the need for continued measurements of interacting climate change factors and moisture mediated responses of ecosystem C metabolism to elevated [CO_2_] and warming [12], [40].

## Supporting Information

Figure S1 **Relationship between midday and daily measurements of ecosystem respiration.** Individual data points reflect pairs of midday measurements and daily sums for a given treatment during diurnal field campaigns conducted in 2007–2010.
(TIF)

Figure S2 **Relationship between midday and daily measurements of ecosystem photosynthesis.** Individual data points reflect pairs of midday measurements and daily sums for a given treatment during diurnal field campaigns conducted in 2007–2010.
(TIF)
