# Supplementary figures and images for: Warming Reduces Carbon Losses from Grassland Exposed to Elevated Atmospheric Carbon Dioxide
Source: PLoS One. 2013 Aug 19;8(8):e71921. doi: 10.1371/journal.pone.0071921 (PMC3747065; doi:10.1371/journal.pone.0071921)

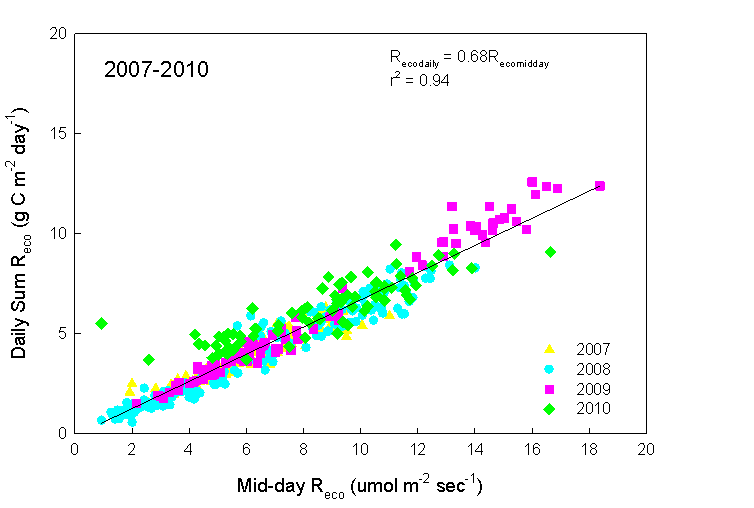

Supplement: Figure S1 — Relationship between midday and daily measurements of ecosystem respiration. Individual data points reflect pairs of midday measurements and daily sums for a given treatment during diurnal field campaigns conducted in 2007–2010. (TIF) [file pone.0071921.s001.tif]

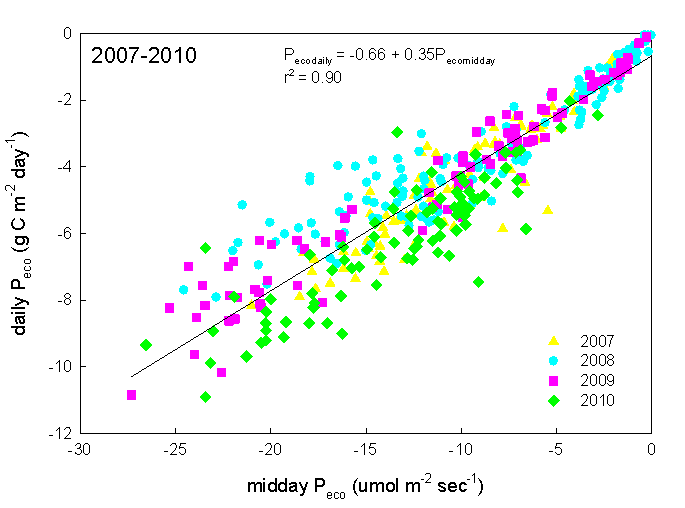

Supplement: Figure S2 — Relationship between midday and daily measurements of ecosystem photosynthesis. Individual data points reflect pairs of midday measurements and daily sums for a given treatment during diurnal field campaigns conducted in 2007–2010. (TIF) [file pone.0071921.s002.tif]
